# On Gout, Rheumatism, Rheumatic Gout, and Sciatica

**Published:** 1864-04

**Authors:** Henry W. Fuller

**Affiliations:** Physician to St. George’s Hospital


### ON GOUT, RHEUMATISM, RHEUMATIC GOUT, AND SCIATICA.

By HENRY W. FULLER, Physician to St. George's Hospital.

[Dr. Fuller considers rheumatic gout a disease *sui generis*—totally distinct from gout, and equally so from rheumatism. It resembles scrofulous inflammation more nearly than rheumatism in its nature. "Rheumatic gout has a history and pathology of its own."]

Pathological research has shown that in the earliest stages of the disease the capsules of the affected joints are distended with fluid, the synovial membrane is thickened and intensely vascular, and vascular tufts or excrescences exist at the margins of the cartilages; that as the disease progresses the fluid is absorbed, the interarticular fibro cartilages are also absorded, and eburnation of the articulating surfaces takes place; that the heads of the bones become enlarged and altered in shape by the occurrence of interstitial absorption in some parts, and of irregular osseous deposits in others; and that foreign bodies of varying consistence and character are often developed, both within and without the joints—bodies which are sometimes cartilaginous, sometimes bony, sometimes attached by longer or shorter pedicles to the synovial membrane, or to the ligamentous structures, and at others are loose within the articulation. It has shown that these changes may take place slowly, without any general febrile disturbance, or any acute local inflammatory action; and on the other hand, that they may be preceded and accompanied by fever, and by pain, heat, and inflammatory swelling of the parts; that the bursæ and sheaths of tendons in the vicinity of the affected joints are prone to be implicated in the mischief, but that neither in the joints nor in the adjacent bursæ or sheaths of tendons are any of the ordinary products of inflammation found—there is no lymph, and no pus, and no urate of soda, as in gout. In other words, it has shown that the characteristic changes which occur in the joints as the result of rheumatic gout take place independently of active inflammation, and that the acute inflammatory action which sometimes precedes or accompanies these structural changes is simply a complication of the disorder, and by no means necessary to its perfect development.

And what are the conditions under which these structural alterations in the joints occur? They are not met with in the robust or vigorous, in well-fed persons with sound constitutions and sedentary habits; they do not arise, like the deposits of urate of soda in gouty men, in connection with excessive indulgence in the luxuries of the table, and defective excretion consequent on a diseased condition of the kidneys. On the contrary, they are more common in women than in men; very frequently arise in persons who lead a temperate life, and are small eaters, and never present themselves in persons who are constitutionally sound, unless they have been subjected to some cause of nervous exhaustion and enfeebled health. Their favorite victims are the offspring of consumptive parents, and especially weakly women—women whose constitution is either originally delicate and unsound, or who, from some cause or another, have fallen into ill health. Amongst men, the most common exciting causes of the disease have appeared to me to be the cachexia, which oftentimes follows excessive venery or syphilis, or the sleeplessness and exhaustion consequent on ill-treated gonorrhœal rheumatism, or the depression resulting from anxiety, or from excessive and long-continued mental exercise, or from over-fatigue or chill in persons of a delicate constitution or scrofulous tendency; whilst in women the disease is often traceable to the cachexia entailed by perversion of the uterine functions. It attacks the girl just arriving at puberty, in whom these functions are ill-performed; it invades the stiffening articulations of the woman who has arrived at that time of life which is marked by the cessation of the monthly periods; it shows itself during the state of debility which follows a miscarriage, or a difficult or protracted labor, more especially when the labor has been accompanied by flooding; and it is a common sequel of over-long suckling.

But whatever the exciting cause of the disease, its primary essential cause is the same in all instances; and although we are unable as yet to point out the precise nature of that cause—although we know little of the morbid chemical actions which take place, and are at a loss to account for the peculiarities in the nutrition of the affected parts by which this form of disease is accompanied, it is impossible to doubt the existence of a special form of constitutional disorder. The history of the complaint, its course and symptoms, and its pathological effects, all indicate the agency of some cause distinct from that which occasions gout or rheumatism. Our inability to demonstrate the nature of the chemical changes in the blood, or, in other words, to prove the formation of a special poison, is not a valid argument against the existence of such a poison. The same line of reasoning would be equally conclusive against the existence of any special form of blood-disorder in small-pox, typhus fever, scarlatina, and pyæmia. The fact is, our means of analysis of organic fluids are at present so imperfect, and we know as yet so little of the influence exerted on the functions of assimilation and excretion by modifications of the nervous power and other similar agencies, that in this, as in other forms of disease, we cannot even offer a reasonable conjecture as to the character of the chemical changes which take place, or as to how those changes are brought about. All that chemistry has as yet enabled us to assert is the bald fact originally pointed out by Dr. Garrod that the blood in these cases does not, like the blood in gout, contain uric or lithic acid.

Thus, then, as there is no very certain mode of diagnosing this disorder, and as, if it is to be treated successfully, its special character must be recognised early in the attack. I will endeavor to bring before you certain facts which will serve as guides to a correct diagnosis.

I would premise that the disease may make its approach either in an acute or in a chronic form. In the latter case, its true character is not likely to be mistaken; but in the former it often resembles an attack of acute rheumatism so closely as to tax our powers of diagnosis to the utmost. There may be heat of the skin and profuse perspiration, furring of the tongue, loading of the urine, acceleration of the pulse, and pain, redness, and swelling of the affected joints—symptoms which, to a greater or less degree, are always attendant on acute rheumatism. But even from the first there are certain peculiarities which ought to excite suspicion as to its nature. The skin, though hot, is less so than acute rheumatism; the perspiration does not possess the peculiar rheumatic odor in any marked degree; the pulse, though quick, is feeble; the tongue is usually less furred; and the local pain and swelling are seldom confined to the knees and other larger joints, but invade the wrist and small joints of the fingers; they are more persistant than the inflammatory swellings of true rheumatism, and they attack a larger number of joints simultaneously.

If the true character of the disorder is overlooked at the first, a few days' observation at the bedside ought to rectify the diagnosis. The symptoms rarely yield to alkalies; the tongue cleans, the heat of the skin subsides, and any slight odor which may have attended the perspiration speedily disappears; but the skin remains constantly bedewed with moisture, and becomes daily more flaccid and less elastic, the pulse gets weaker, and the pain and swelling of the smaller joints assume a more prominent aspect. The inflammation, however, though continuing so obstinately, is not so acute, and does not appear to threaten the integrity of the joint, as true rheumatic inflammation does under similar circumstances. When true rheumatism fixes obstinately on a joint, the fear of perminent mischief and anchylosis of the joint at once presents itself to the mind. The inflammation of the other joints subsides, but the pain and swelling in the one joint increase daily; and it is obvious to the merest tyro in medicine that if that joint be not kept motionless, and leeches, blisters, and fomentations, or mercurial ointment applied, anchylosis of the joint is the most favorable issue which can be expected. But it is otherwise in respect to the inflammation of the joints which accompanies rheumatic gout. Rarely, indeed, in the acute form of the disease, is the inflammation confined to one joint; on the contrary, three or four, or even a larger number, of the joints remain affected throughout. There is not the same heat, or redness, or tenderness of the effected joints; the fear of adhesive or suppurative mischief does not arise; the application of a splint, and of leeches and blisters, does not suggest itself; and although the joints may remain permanently enlarged and distorted, they do not become anchylosed.

When the disease makes its approach more slowly, and assumes from the first a non-acute or chronic form, its features are much more distinctive. The patient feels weak, languid, and uncomfortable; she is oftentimes chilly, but nevertheless perspires on the slightest exertion; the appetite is capricious, the pulse feeble, the urine often pale and clear, and the spirits are much depressed. Up to this time probably there may have been no swelling of the joints, and possibly no wandering pains in the limbs, so that no suspicion is entertained as to the nature of the impending mischief. The ill-health is attributed to the effect of a mercurial course, to the drain resulting from an excessive flow of the monthly courses, to profuse leucorrhœa, to amenorrhœa, or to one of the many causes which are productive of ill-health, and which may have been present in the particular case in question. But after a longer or shorter period, some pain or stiffness is perceived in one or more of the joints. Not unfrequently a knuckle becomes stiff and swollen for weeks or months before any other joint is effected; and even though the knees or other of the larger joints be enlarged, the knuckles rarely escape. They are seldom red, inflamed, or very tender to the touch; on the contrary, they are relieved by gentle friction, and will often derive benefit even from tolerably active rubbing. Effusion within the joint is the principal cause of their enlargement; but the bursæ and sheaths of tendons around the joint are also implicated, and are felt as circumscribed swellings. Moreover, the mischief is seldom confined to the immediate vicinity of the joints, but the sheaths of tendons may be felt hard and swollen in the palms of the hands and in other parts more or less remote from the primary seat of inflammation.

In the more advanced stages of the chronic form of the disorder, the peculiarities of the case become even more apparent. Depression of spirits is a prominent symptom; the constant clammy moistness of the skin is quite characteristic; the extraordinary number of joints implicated in the mischief is unlike what is observed in any variety of true rheumatism; and the form of the articular swelling is such as cannot possibly be confounded with the effects of rheumatism. It is obviously due, in great measure at least, to enlargement of the extremities of the bones themselves, and not merely to effusion within their capsules, or to the thickening of the surrounding structures. Thus a material alteration occurs in the form, and oftentimes in the direction of the joints. The fingers, for instance, are drawn towards the ulnar or outside of the hand, and take a permanently oblique direction; whilst the enlarged and partly dislocated extremities of the bones, more especially of the metacarpal bones, project in every variety of form, and constitute the nodosities which have been described by Dr. Haggarth in his "Clinical History of Disease."

Thus, then, to sum up the principal facts which have a practical bearing on the treatment of the disease, it may be stated: 1st. That the malady originates in mal-nutrition, resulting not unfrequently from some hereditary infirmity of constitution, but sometimes in connexion with cachexia induced by a variety of causes which exhaust the nervous system. 2d. That the local changes to which it gives rise are essentially distinct from those produced by active inflammation, and more nearly resemble the results which might be expected from a slow perversion of nutrition; indeed, a similar tendency to the formation of exuberant osseous growths around the joints whilst the articular textures within are suffering destruction and decay is observed in malignant disease of the joints, and in various strumous affections of the joints, both of which are connected with a constitutional taint. 3d. That whether in an acute or in a chronic form, the malady is one and the same, due to the same cause, connected with a similar failure of tone in the system, and productive of similar changes in the joints; the only difference observable between the results in the acute and chronic cases respectively being that in the former they occur more rapidly than in the latter.

If this view as to the nature of the disorder is correct,—and its whole history leaves little room for doubt on the matter,—it follows that any treatment to be successful must have for its object the sustentation of the general health and the restoration of tone to the system. Whilst this is being effected, means may be taken to subdue the local irritation of the joints, and thus to mitigate our patient's suffering; but the primary object must be to improve the health, and so to check the continuance of those actions on which the enlargement and distortion of the joints depend. The remedies which are most serviceable in rheumatism and gout are of little avail in this form of disease. Colchicum, iodide of potassium, guaiacum, hot baths, vapor baths and other similar remedies, if prescribed with a view to eradicate the disease, prove mischievous rather than beneficial. They depress and enervate the patient, who is already low and exhausted; and thus they serve to establish the disorder which they were given expressly to get rid of. In private no less than in hospital practice the mischievous results which follow this mode of treatment almost daily force themselves on my attention. In short, if the remedies above named are to be employed at all in the treatment of rheumatic gout, they should be used cautiously as alteratives in conjunction with tonics, and should not be administered as agents to be relied upon for the cure of the disease. The more I have seen of this form of the disorder, the more thoroughly have I discarded the views which, in common with other medical men, I formerly entertained respecting its treatment, and the more completely have I learned to trust to tonics and occasional alteratives. In the acute stage of the disorder it may be necessary for a few days to administer alkalies and alterative doses of blue-pill or calomel, and to restrict the diet to broth or beef-tea; but when once the true nature of the malady has declared itself, I believe that in the majority of instances the more successful plan, notwithstanding the acute character of the symptoms, is to administer bark or quinine in combination with small doses of alkalies, and as soon as possible to interpose and check the continuance of the enfeebling, clammy perspiration by means of a cold shower-bath or the dripping-sheet. Indeed, whether the disease be in an acute or in a chronic form, the general state of the system and the ever-varying condition of the secretions are the only rational guides to treatment. If, as often happens when the disease is chronic, the secretions are tolerably regular and healthy, if the bowels are acting daily, and the alvine dejections are of a natural color, if the urine is clear, and remains so on cooling, and if the skin is neither dry nor damp and clammy, the most effectual remedies are bark, quinine, strychnine, iron, in its different forms, cantharides, arnica, sarsaparilla, the mineral acids, and cod-liver oil; and they must be given in doses proportioned to the amount of depression they have to counteract; further, their action must be assisted by fresh air and exercise, change of scene, and a generous diet: meat twice or three times daily, with a full allowance of porter or ale, and wine, are essential adjuncts to the treatment. On the other hand, if the motions are pale, calomel or blue-pill must be given as alteratives; if the urine is loaded with lithates and the bowels are torpid, these secretions must be regulated in the ordinary way by the exhibition of purgatives and alkalies; the diet at the same time must be more or less restricted, and malt liquor prohibited. But even in these cases care must be taken not to depress the patient; and while brandy, or gin, or whisky is substituted for the malt liquor and wine, an endeavor should be made to discover some nutritious food which the patient can digest and assimilate. If the skin is clammy, and the shock of cold water is followed by reaction and warmth of the surface, a cold shower-bath or the dripping-sheet should be employed daily, for nothing tends so powerfully to stimulate the capillary circulation and restore the tone of the system.

You will always do well to inquire whether your patient ordinarily enjoys better health in winter or in summer, in cold weather or in warm,—as the reply you obtain will serve as a tolerable trustworthy criterion as to whether she is likely to be benefited by cold water. When a patient who is suffering from rheumatic gout tells you that she is usually stronger and more vigorous in cold weather than in hot, you may confidently recommend the daily use of a shower-bath. It will brace and stimulate her, and will contribute as largely to her recovery as any medicine you can administer internally. There is nothing more remarkable in the whole range of therapeutics than the rapidity of the improvement sometimes observed under the influence of this remedy. Even when a patient tells you that she is not much affected by temperature, but that she is often chilly and dreads cold water, I would urge you not to be deterred from having recourse to its assistance. The chilliness in such a case is dependent on the vitiated condition of her blood, and not on any innate delicacy or any deficiency in her power of resistance to cold: consequently the shower-bath, by imparting a stimulus to the system, will conduce to the better performance of the various functions of the body, and so to an improvement in the condition and circulation of the blood, and to a cessation of the sense of chilliness. If you are not consulted until the patient has been greatly reduced by illness, and has a feeble pulse, and scarcely sufficient power to enable her to rally from the shock of the shower-bath, you may order the dripping-sheet, as you have seen me do, with excellent results, under similar circumstances. The shock is somewhat less, and so also is the chilling effect on the system. But I would have you bear in mind that it is impossible *a priori* to determine whether reaction will take place after the shock of cold water. Sometimes a person whose circulation is so weak as to render it improbable that she would be able to withstand it, will feel refreshed and invigorated by it, and will glow with warmth in a few minutes afterwards; whereas, another person, apparently stronger and less reduced by illness, will be chilled and exhausted by it, and will suffer from cold the whole day. One or two trials, however, will settle this question, and the bath is such an important agent in the treatment of these cases that the matter ought never to be allowed to remain undecided. The only cases in which its effect is doubtful are those in which the patients, even when in health, were always better in summer than in winter, and have always suffered from cold. In these cases, if the clamminess of the skin appears to require the use of the cold shower-bath, a small quantity only of cold water should be employed. The patient, when taking it, should stand in hot water, and a cotton sheet should be thrown round her as soon as she steps out of the bath. With these precautions there are few persons who cannot withstand the shock of the water, and as few who will not benefit by it.

Another point is relative to the use of a cold douche, and the means of obtaining one. A few jugs of water poured on the affected joint are of little or no service in these cases: the force of the water is not sufficiently great, nor is its action sufficiently sustained. In none of the London or provincial hospitals with which I am acquainted, nor in most of the towns in the Un Kingdom, is a good douche-bath to be found, and therefore if we wish to avail ourselves of this valuable agent in the treatment of stiffened joints we must either send our patients to a hydropathic establishment, where this species of bath is admirably arranged, or extemporize one for ourselves at the residences of our patients. Fortunately, it is not difficult in most houses to arrange an efficient douche-bath for the knees and ankles—the parts to which this description of bath is specially applicable. All that is required is a large tap, communicating with a cistern of water placed at a considerable elevation above it. Two yards of India-rubber tubing affixed to the tap will suffice to carry the stream of water towards the affected joint, and a large, empty bath will receive it as it flows. You may think this a trivial matter for me to dilate upon, but if you are really in earnest at your work, and are aiming at the attainment of proficiency in relieving human suffering, you will not despise the smallest matters which can contribute towards the object you have in view.—*Lancet, Sept.* 26, 1863, *p.* 355.

